# Differential relationship between waist circumference and mortality according to age, sex, and body mass index in Korean with age of 30–90 years; a nationwide health insurance database study

**DOI:** 10.1186/s12916-018-1114-7

**Published:** 2018-08-10

**Authors:** Geum Joon Cho, Hye Jin Yoo, Soon Young Hwang, Jun Choi, Kyu-Min Lee, Kyung Mook Choi, Sei Hyun Baik, Sung Won Han, Tak Kim

**Affiliations:** 1 Department of Obstetrics and Gynecology, Korea University Guro Hospital, Korea University College of Medicine, Seoul, Republic of Korea; 2 Division of Endocrinology and Metabolism, Department of Internal Medicine, Korea University Guro Hospital, Korea University College of Medicine, Seoul, Republic of Korea; 3 Department of Biostatistics, College of Medicine, Korea University, Seoul, Republic of Korea; 4 School of Industrial Management Engineering, Korea University, Seoul, Republic of Korea; 5 Department of Obstetrics and Gynecology, Korea University Anam Hospital, Korea University College of Medicine, 73, Inchon-ro, Seongbuk-gu, Seoul, 02841 Republic of Korea

**Keywords:** Obesity, Mortality, Body mass index, Waist circumference

## Abstract

**Background:**

A recent concept is that obesity, assessed by body mass index (BMI), is not always a sign of poor health. Thus, in order to use obesity metrics in clinical decision making, it is important to clarify the relationship between waist circumference (WC), a proxy for abdominal obesity, and mortality.

**Methods:**

Data were used from 8,796,759 subjects aged between 30 and 90 years, who had participated in the Korea National Health Screening Examination between January 1, 2009 and December 31, 2009 and survived at least 1 year post screening. Data from a mean follow-up time of an additional 5.3 years (time at risk) were analyzed for the relationship between WC and mortality according to age, sex, and BMI category.

**Results:**

An increased WC of more than 90 cm in men and 85 cm in women showed a definite negative influence on mortality. However, the detailed relationship between WC and mortality was J-shaped or U-shaped according to age, sex, and BMI category. In the normal BMI group, the optimal WC range with the lowest mortality was < 70 cm in men and 70–75 cm in women, whereas in obese individuals a WC between 80 and 90 cm in men and 75 and 85 cm in women showed the lowest mortality. The association between increased WC and higher mortality tended to be more obvious in normal-weight women than in normal-weight men or obese women. Furthermore, in normal-weight and obese women, the effect of increased WC on mortality was more critical for subjects aged < 60 years rather than those aged ≥ 60 years.

**Conclusions:**

Abdominal obesity, as measured by WC, showed a significant negative association on mortality, and its association with mortality was different according to age, sex, and BMI category. Therefore, WC should be considered in the assessment of obesity-related health risks, and individualized cut-off points for the definition of a healthy WC according to age, sex, and BMI category are necessary.

**Electronic supplementary material:**

The online version of this article (10.1186/s12916-018-1114-7) contains supplementary material, which is available to authorized users.

## Background

Recent epidemiological data have shown that the lowest risk for mortality is observed in overweight and mildly obese individuals, not those in the normal weight group [1–3]. This phenomenon, in which overweight and obese individuals exhibit a better prognosis than leaner subjects, is called the obesity paradox [4]. However, most studies supporting the obesity paradox utilized body mass index (BMI) for the definition of obesity, which does not discriminate between lean mass and fat mass [5].

Waist circumference (WC) is strongly correlated with visceral adipose tissue, the body composition component that most strongly causes metabolic disturbances [6]. Janssen et al. [7] reported that WC in combination with BMI is a better predictor for abdominal fat than BMI alone, mainly by explaining an additional variation of visceral fat. Several studies have shown that WC has a positive association with all-cause mortality after adjusting for other covariates including BMI [8–10]. Body composition continuously changes with age, and these changes are different between men and women [11]. However, there have been very limited studies that focus on the association between WC and mortality according to age and sex. Generally, men start to lose muscle mass at the end of their fifth decade; women show a similar decline in lean mass, but gain greater fat mass [12]. Women exhibit a more peripheral fat distribution in early adulthood, whereas postmenopausal women and men show a more central fat accumulation [13]. Nevertheless, a uniform cut-off value of unhealthy WC regardless of age both in men and women can be problematic. Furthermore, Asians have a larger amount of metabolically active visceral fat than Caucasians at the same BMI [14]. As a result, Asians tend to develop metabolic deterioration with relatively lower degrees of obesity and might have a differential relationship between WC and mortality compared to other ethnic groups. Until now, there has been no prospective cohort study to examine the specific effects of age, sex, and BMI on the relationship between WC and mortality in Asians.

Therefore, to assess the relationship between WC and mortality according to age, sex, and BMI, we compared relative mortality risk according to WC stratified by age, sex, and BMI category using the large-scale National Health Screening Examination (NHSE) database. Moreover, we tried to determine an optimal WC cut-off range for Koreans with the lowest mortality risk according to BMI category. In this study, to exclude the potential confounding effect of concealed baseline diseases on mortality, we calculated a hazard ratio (HR) of mortality after adjusting for the Charlson Comorbidity Index (CCI), a valuable scoring system of underlying comorbidity [15].

## Methods

### Health care delivery system in Korea

In Korea, 97% of the population is obligated to enroll in the Korea National Health Insurance (KNHI) program; the remaining 3% is covered by the Medical Aid Program. Therefore, the KNHI claims database contains information on all claims for approximately 50 million Koreans, and nearly all information about the extent of a disease can be obtained from this centralized database except for procedures not covered by insurance, such as cosmetic surgery. As part of the National Health Insurance Service (NHIS) healthcare system, all the nationwide population aged ≥ 40 or regular workers and their dependents are invited to participate in a biannual NHSE free of charge.

### Study population

**Fig. 1:**
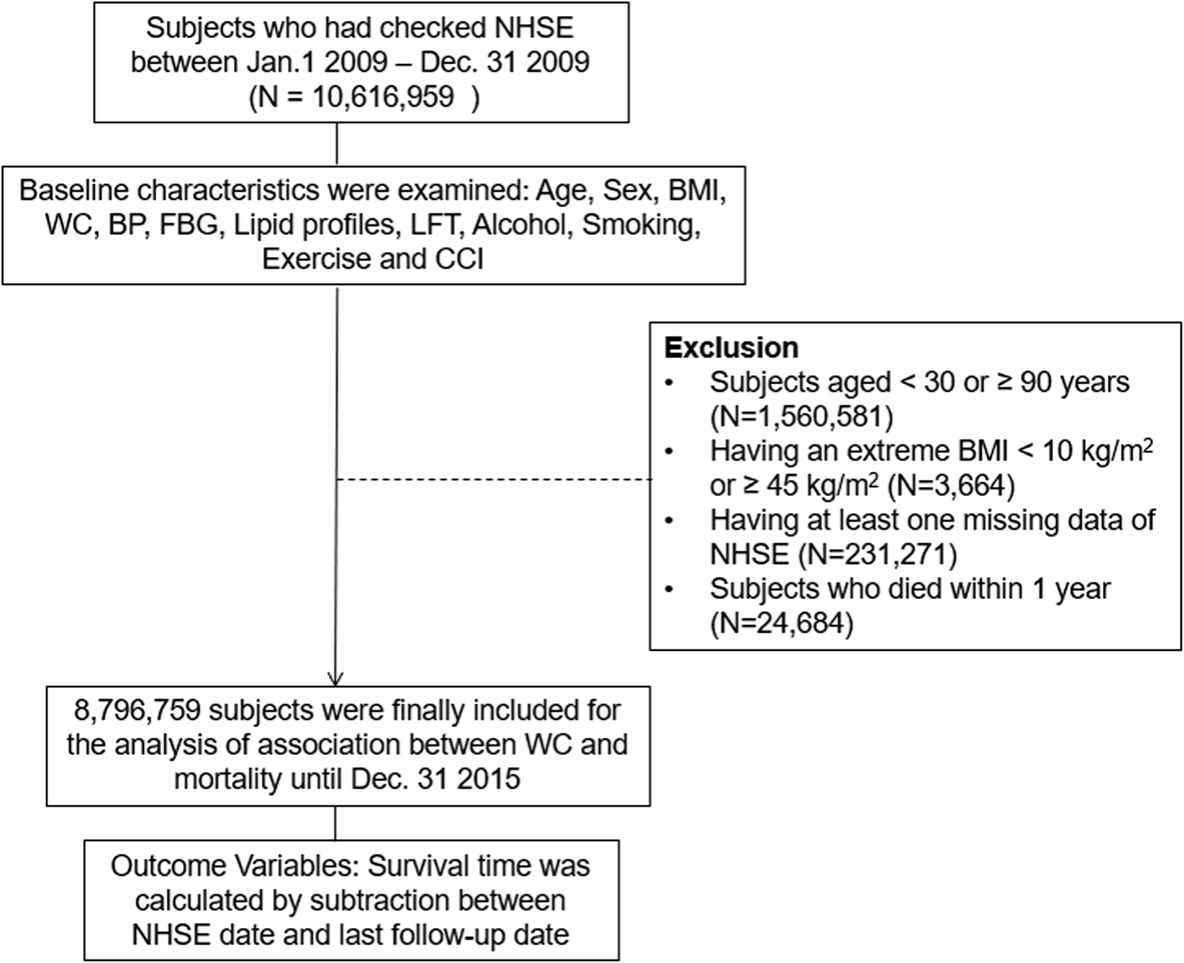
Flow chart of study diagram. *BMI* body mass index, *WC* waist circumference, *BP* blood pressure, *FBG* fasting blood glucose, *LFT* liver function test, *CCI* Charlson Comorbidity Index

A flow chart of patient enrollment is shown in Fig. 1. Using the NHIS database, we identified all subjects who had participated in the NHSE between January 1, 2009 and December 31, 2009. Among these 10,616,959 people, subjects aged < 30 or ≥ 90 years (*N* = 1,560,581), who had an extreme BMI < 10 kg/m^2^ or ≥ 45 kg/m^2^ (*N* = 3664), or had at least one missing value in the studied variables (*N* = 231,271), or who died within 1 year (*N* = 24,684) were excluded. As a result, 8,796,759 subjects were followed up for their death until December 31, 2015, which had been registered to the National Death Registry. The mean follow-up duration for death was 5.3 years (time at risk). This study was approved by the Institutional Review Board of Korea University Medical Center (KUGH16284-001).

### Measurement of baseline characteristics

The NHSE consists of a health interview and health examination. The health interview includes questions regarding demographic, socioeconomic, and lifestyle status. Data for the following covariates were obtained: age, smoking status, alcohol consumption, and exercise level. Smoking status was classified as current smoker, past smoker, or never smoker based on the answers to “Have you ever been a smoker?” and “If yes, do you smoke currently?” Alcohol was categorized according to the weekly frequency of alcohol consumption: no alcohol consumption, 1–2 servings/week, or 3 or more servings of alcohol per week. Exercise was categorized according to the weekly frequency of full-body, sweat-inducing exercise: no exercise, 1–2 exercise sessions per week, or ≥ 3 exercise sessions per week.

The health examination included the calculation of BMI in kilograms per meter squared using height and weight measurements. WC was measured at the narrowest point between the lower border of the rib cage and the iliac crest during minimal respiration. For analyses, WC was categorized into 5-cm increments: < 70, 70– <75, 75– <80, 80– <85 (reference for women), 85– <90 (reference for men), 90– <95, 95– <100, and ≥ 100 cm. The reference WC was selected as the category just below the Korean criteria for abdominal obesity [16]. Blood pressure (BP) was measured using a standard mercury sphygmomanometer. All blood samples were obtained after a minimum fast of 8 h. The levels of fasting blood glucose (FBS), total cholesterol, triglycerides (TG), high-density lipoprotein cholesterol (HDL-C), low-density lipoprotein cholesterol (LDL-C), aspartate aminotransferase (AST), and alanine aminotransferase (ALT) were measured with a Hitachi 747 Autoanalyzer (Hitachi Instruments Inc., Tokyo, Japan) using enzymatic methods.

In this study, comorbidities were measured by the CCI. The CCI was defined based on 19 chronic diseases using the International Classification of Diseases, 10th edition (ICD-10) codes [17] including diabetes with diabetic complications, cerebrovascular diseases, congestive heart failure, chronic pulmonary disease, mild and severe liver disease, hemiplegia, renal disease, leukemia, lymphoma, metastatic tumor, and acquired immunodeficiency syndrome (AIDS) [18]. In this study, the CCI was classified into two groups with score 0, indicating no comorbidity, or 1, meaning at least one or more comorbidities.

### Outcomes

The primary study outcome was all-cause mortality. All-cause mortality was defined as death status in the KNHI database, which was linked to the National Death Registry using unique resident registration numbers. Survival time was calculated by subtraction between NHSE date and last follow-up date, the same value with subtraction between last follow-up age and baseline age.

### Statistical analysis

**Fig. 2:**
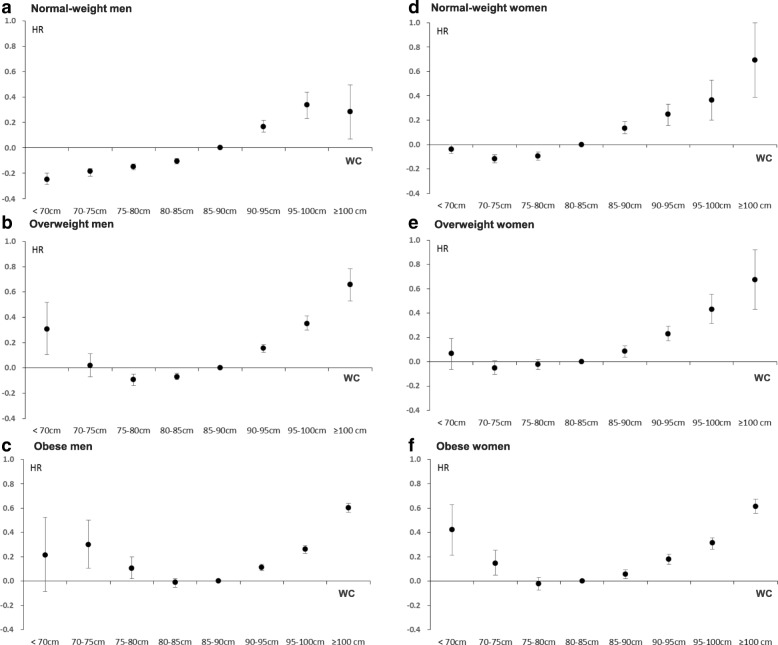
Hazard ratios for mortality according to WC interacted by BMI categories in men (**a**–**c**) and women (**d**–**f**) after adjusting for age, BMI (as a continuous variable), alcohol, smoking and exercise status, Charlson Comorbidity Index, and laboratory tests. The *p* values for interactions between WC categories (8 groups) and BMI categories (4 groups) on mortality were < 0.001 in both men and women. *HR* hazard ratio, *WC* waist circumference

**Fig. 3:**
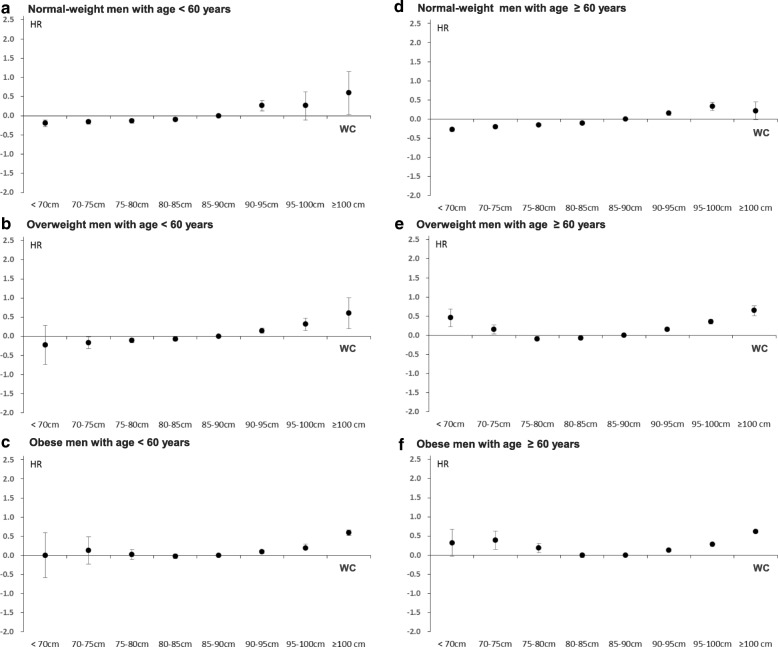
Hazard ratios for mortality according to WC interacted by BMI categories in men aged < 60 years (**a**–**c**) and those aged ≥ 60 years (**d**–**f**) after adjusting for age, BMI (as a continuous variable), alcohol, smoking and exercise status, Charlson Comorbidity Index, and laboratory tests. The *p* values for interactions between WC categories (8 groups) and BMI categories (4 groups) on mortality were 0.044 in men aged < 60 years and < 0.001 in those aged ≥ 60 years

**Fig. 4:**
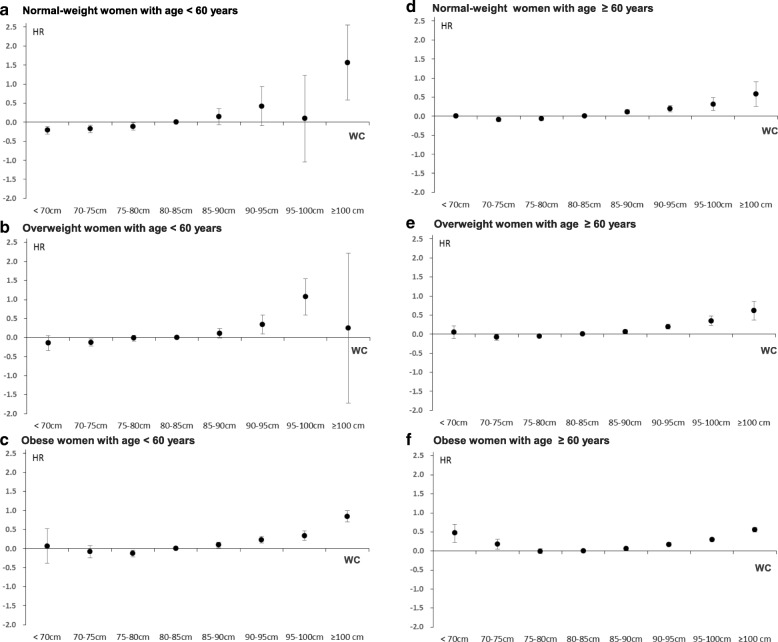
Hazard ratios for mortality according to WC interacted by BMI categories in women aged < 60 years (**a**–**c**) and those aged ≥ 60 years (**d**–**f**) after adjusting for age, BMI (as a continuous variable), alcohol, smoking and exercise status, Charlson Comorbidity Index, and laboratory tests. The *p* values for interactions between WC categories (8 groups) and BMI categories (4 groups) on mortality were 0.458 in women aged < 60 years and < 0.001 in those aged ≥ 60 years

Continuous and categorical variables are expressed as mean ± standard deviation (SD) and percentages, respectively. Clinical and biochemical characteristics were compared among two groups using an independent two-sample *t* test for continuous variables and Pearson’s chi-square test or Fisher’s exact test for categorical variables (Table 1 and Additional file 1: Table S3). Clinical and biochemical characteristics were compared among three or more groups using independent one-way analysis of variance (ANOVA) for continuous variables and Pearson’s chi-square test or Fisher’s exact test for categorical variables. A contrast test in ANOVA for continuous variables and the Mantel-Haenszel chi-square test for categorical variables were used to calculate *p* values for linear trends (Additional file 1: Tables S1 and S2). Cox proportional hazards models were used to estimate the adjusted HRs and 95% confidence interval (CIs) of mortality according to baseline WC category interacted by BMI category in men and women, respectively. In this analysis, BMI category was classified into the four groups according to the obesity guideline for an Asian population: low weight < 18.5 kg/m^2^, normal weight 18.5 ≤ BMI < 23 kg/m^2^, overweight 23 ≤ BMI < 25 kg/m^2^, and obesity BMI ≥ 25 kg/m^2^ [16]. WC category was classified into 5-cm increments: < 70, 70–< 75, 75–< 80, 80– <85 (reference for women), 85– <90 (reference for men), 90– <95, 95– <100, and ≥ 100 cm. In the Cox regression analysis, the time at risk was set to be started after 1 year of the NHSE date, and the censoring/death was monitored until December 31, 2015. To examine the differential effects of the WC categories (8 groups) on mortality according to the BMI categories (4 groups), we tested the interaction between WC categories and BMI categories by adding interaction terms into the Cox proportional hazards models. To minimize confounding effects, covariates of baseline age, alcohol, smoking and exercise status, BMI (as a continuous variable), FBS, total cholesterol, HDL-C, LDL-C, AST, ALT, and comorbidities, assessed by the CCI, were subjected to statistical adjustments (Table 2 and Fig. 2). We then performed Cox proportional hazard analysis stratified by age groups (< 60 or ≥ 60 years) in men and women using the same covariates and interaction terms (Additional file 1: Table S4 and Figs. 3, 4). All tests were two-sided, and *p* values < 0.05 were considered statistically significant. Statistical analysis was performed using SAS 9.2 (SAS Institute, Cary, NC, USA).

**Table 1:** Baseline characteristics of the study subjects

	Men		Women		*p* value
	(*N* = 4,845,863)		(*N* = 3,950,896)		
Age (years)	47.8	± 12.2	51.6	± 11.9	< 0.001
BMI (kg/m^2^)	24.2	± 3.0	23.6	± 3.2	< 0.001
WC (cm)	84.0	± 7.6	77.4	± 8.6	< 0.001
Systolic BP (mm Hg)	125.2	± 14.4	121.3	± 15.9	< 0.001
Diastolic BP (mm Hg)	78.4	± 9.8	75.1	± 10.2	< 0.001
Fasting glucose (mg/dL)	100.5	± 26.8	96.5	± 22.0	< 0.001
TC (mg/dL)	196.5	± 41.3	199.5	± 42.4	< 0.001
HDL-C (mg/dL)	53.3	± 31.6	59.2	± 34.8	< 0.001
LDL-C (mg/dL)	117.3	± 135.9	121.0	± 98.6	< 0.001
AST(IU)	28.0	± 27.6	23.7	± 22.2	< 0.001
ALT(IU)	30.3	± 31.4	21.0	± 23.1	< 0.001
Smoking history (%)					
Never	30.0		95.4		< 0.001
Past	26.9		1.6		< 0.001
Current	43.1		3.0		< 0.001
Alcohol (%)					
0 serving/week	32.9		78.4		< 0.001
1–2 servings/week	44.6		18.2		< 0.001
≥ 3 servings/week	22.5		3.4		< 0.001
Exercise (%)					
0 session/week	51.3		63.2		< 0.001
1–2 sessions/week	28.4		18.5		< 0.001
≥ 3 sessions/week	20.3		18.3		< 0.001
Charlson Comorbidity Index score: 0 (%)	22.9		13.0		< 0.001

Data are expressed as the mean ± SD or percentage. *p* values were calculated by an independent two-sample *t* test or Pearson’s chi-square test. For Charlson Comorbidity Index score, 0 means that the subject is without any comorbidity

*BMI* body mass index, *WC* waist circumference, *BP* blood pressure, *TC* total cholesterol, *HDL-C* high-density lipoprotein cholesterol, *LDL-C* low-density lipoprotein cholesterol, *AST* aspartate aminotransferase, *ALT* alanine aminotransferase

**Table 2:** Hazard ratios (95% confidence intervals) for mortality according to WC categories by BMI categories, stratified by sex

	BMI < 18.5 kg/m^2^	18.5 ≤ BMI < 23	23 ≤ BMI < 25	BMI ≥ 25
Men				
WC (cm)				
< 70	0.96 (0.83, 1.10)	0.78 (0.75, 0.82)	1.36 (1.11, 1.68)	1.24 (0.92, 1.69)
70–75	0.98 (0.85, 1.13)	0.83 (0.80, 0.85)	1.02 (0.93, 1.12)	1.35 (1.11, 1.65)
75–80	1.10 (0.95, 1.27)	0.86 (0.84, 0.88)	0.91 (0.87, 0.95)	1.11 (1.02, 1.22)
80–85	1.12 (0.85, 1.48)	0.90 (0.88, 0.92)	0.93 (0.91, 0.96)	0.99 (0.95, 1.02)
85–90	1	1	1	1
90–95	0.98 (0.73, 1.32)	1.18 (1.13, 1.24)	1.17 (1.13, 1.20)	1.12 (1.09, 1.15)
95–100	1.22 (0.74, 2.00)	1.40 (1.26, 1.55)	1.42 (1.35, 1.51)	1.30 (1.26, 1.34)
≥ 100	1.34 (0.43, 4.18)	1.33 (1.07, 1.64)	1.93 (1.70, 2.19)	1.83 (1.76, 1.90)
Women				
WC (cm)				
< 70	0.72 (0.64, 0.81)	0.96 (0.93, 1.00)	1.07 (0.94, 1.21)	1.53 (1.24, 1.88)
70–75	0.78 (0.70, 0.88)	0.89 (0.86, 0.92)	0.95 (0.90, 1.01)	1.16 (1.05, 1.29)
75–80	0.87 (0.76, 0.99)	0.91 (0.88, 0.94)	0.98 (0.94, 1.02)	0.98 (0.93, 1.03)
80–85	1	1	1	1
85–90	0.88 (0.71, 1.09)	1.14 (1.09, 1.21)	1.09 (1.04, 1.14)	1.06 (1.02, 1.10)
90–95	0.88 (0.63, 1.25)	1.28 (1.17, 1.39)	1.26 (1.19, 1.34)	1.20 (1.15, 1.25)
95–100	0.19 (0.06, 0.59)	1.44 (1.22, 1.70)	1.54 (1.37, 1.74)	1.37 (1.30, 1.43)
≥ 100	0.85 (0.32, 2.28)	2.00 (1.47, 2.72)	1.96 (1.54, 2.51)	1.85 (1.75, 1.96)

Data are expressed as the hazard ratio (95% confidence interval)

BMI category was classified into the four groups: low weight < 18.5 kg/m^2^, normal weight 18.5 ≤ BMI < 23 kg/m^2^, overweight 23 ≤ BMI < 25 kg/m^2^, and obesity BMI ≥ 25 kg/m^2^

The hazard ratios (95% confidence intervals) were calculated by the Cox proportional hazards model with baseline age, alcohol, smoking and exercise status, BMI (as a continuous variable), BMI categories (4 groups), WC categories (8 groups), interaction between BMI categories and WC categories, FBS, total cholesterol, HDL-C, LDL-C, AST, ALT, and comorbidities, assessed by CCI

## Results

### Baseline characteristics of the study population

Among the total 8,796,759 participants, 4,845,863 were men, and 3,950,896 were women. More detailed baseline characteristics of the study participants are described in Table 1. When WC was stratified into 5-cm increments from 70 to 100 cm, BMI, systolic BP, diastolic BP, FBS, AST, ALT, LDL-C, and total cholesterol levels were significantly increased with increasing WC, whereas HDL-C level and the portion of subjects with CCI score 0 was significantly decreased with increasing WC (Additional file 1: Table S1). When BMI was classified to the four categories and age was subdivided into the two groups, the baseline characteristics also significantly changed according to the increase of BMI or age. The detailed baseline characteristics of these groups are displayed in Additional file 1: Tables S2 and S3.

### Association of WC with mortality according to the BMI categories in men and women

During the median 5.3 years of follow-up (time at risk), 222,087 deaths occurred (2.5% of the cohort). The HRs of mortality according to WC categories are described in Table 2. Except for the low BMI category, all the subjects with WC more than the current cut-off points for abdominal obesity (men ≥ 90 cm, women ≥ 85 cm) showed a significant increased risk for mortality after adjusting other covariates (Table 2, Fig. 2). Furthermore, the association between WC categories and mortality showed varying relationships according to BMI categories (*p* value for interaction between WC categories and BMI categories < 0.001); in normal-weight and overweight women, the association between WC and mortality exhibited J-shaped relationships (Fig. 2d and e), whereas in overweight men and obese subjects, the relationship was U-shaped (Fig. 2b, c, and f). In the normal-weight men, the lowest HRs for mortality were observed in the subjects with a WC of < 70 cm (HR 0.78; 95% CI 0.75–0.82). In normal-weight women, the lowest HRs were for those with a WC of 70–75 cm (HR 0.89; 95% CI 0.86–0.92) (see Table 2). Normal-weight women showed a unified increased HR according to the increase of WC more than the reference range (Fig. 2d), whereas in normal-weight men the monotonic increase of HR was not observed; the HR for mortality of WC between 95 and 100 cm was larger than that for WC more than 100 cm in men (Fig. 2a). In overweight subjects, the lowest HRs for mortality were observed for men with a WC of 75–80 cm (HR 0.91; 95% CI 0.87–0.95) and for women with a WC of 70–75 cm (HR 0.89; 95% CI 0.86–0.92) (see Table 2). The overweight men with WC < 70 cm showed a significant increased risk for mortality (Fig. 2b), which was not the case for women (Fig. 2e). In obese subjects, the lowest HRs for mortality were observed in the subjects with WC between 80–90 cm in men and 75–85 cm in women (Table 2). In obese group, the subjects with WC less than 80 cm in men and 75 cm in women showed a more increased risk for mortality than the reference group (Fig. 2c and f).

### Association of WC with mortality according to the BMI categories in men and women stratified by age < 60 or ≥ 60 years

In the normal-weight men and women aged < 60 years (Figs. 3a and 4a), the HRs for mortality in the subjects with WC ≥ 100 cm when compared to the reference group (men: HR 1.81; 95% CI 1.03–3.19, women: HR 4.76; 95% CI 1.78–12.82, Additional file 1: Table S4) were more profound than those for subjects aged ≥ 60 years (Figs. 3d and 4d) (men: HR 1.24; 95% CI 0.98–1.56, women:HR 1.78; 95% CI 1.29–2.46, Additional file 1: Table S4). Similarly, in obese women aged < 60 years, the HRs for mortality in the subjects with WC ≥ 85 cm when compared to the reference group were more obvious than those of obese women with age ≥ 60 years (Fig. 4c and f, Additional file 1: Table S4). The overweight men and obese subjects aged ≥ 60 years showed a U-shaped association between WC and mortality (Fig. 3e, f and Fig. 4f). Except for these groups, all the remaining groups exhibited J-shaped relationships between WC and mortality (Fig. 3a–d and Fig. 4a–e).

## Discussion

This study confirmed that abdominal obesity, defined as WC ≥ 90 cm in men and ≥ 85 cm in women in Koreans, has a definite negative influence on mortality. The association of WC with mortality and the optimal WC range with the lowest mortality were quite different according to age, sex, and BMI category. The association between increased WC and higher mortality tended to be more obvious in normal-weight women than in normal-weight men or obese women. Furthermore, in normal-weight and obese women, the effect of increased WC ≥ 100 cm on mortality was more critical for subjects aged < 60 years than for those aged ≥ 60 years.

The obesity paradox is a very controversial concept, because it can lead people to disregard the unhealthy metabolic consequences of excess adipose tissue. Also, the obesity paradox must not be confused with the limitation of BMI. Although BMI is the most commonly used parameter for identifying obesity due to its simplicity, it cannot fully reflect the risk of obesity-related metabolic complications and death [5]. In contrast to the findings from general obesity as measured using BMI, central obesity measured using WC was more consistently related to higher mortality. In a meta-analysis of six studies including 15,923 people with coronary heart disease, central obesity defined as having the highest tertiles of waist-to-hip ratio (WHR) or WC was associated with increased mortality (HR 1.70; 95% CI 1.58–1.83) when compared to the reference group having the lowest tertiles of WHR or WC, whereas the group with the highest tertile of BMI was inversely associated with mortality (HR 0.64; 95% CI 0.59–0.69) [19]. The present study showed that, for Koreans, increased WC that was higher than current cut-off points for abdominal obesity, ≥ 90 cm in men and ≥ 85 cm in women, was significantly associated with higher mortality after adjusting for BMI and other covariates. After adjusting for BMI, a larger WC reflected higher visceral fat, a well-known risk factor for cardiometabolic disturbances and certain cancers [20]. Inflammation of visceral adipose tissue mediates metabolic disturbances regardless of generalized obesity [21], and excess adipose tissue increases free fatty acid release, which promotes cellular proliferation and tumor growth [22]. The present study showed that in the normal-weight individuals the optimal cut-off range of WC with the lowest mortality was a much lower value than the current cut-off point for defining abdominal obesity in Korea [16]. In the normal-weight group, the optimal cut-off range showing the lowest mortality was < 70 cm in men and 70–75 cm in women, whereas in the obese group, this cut-off range was 80–90 cm in men and 75–85 cm in women, suggesting the necessity of a BMI-category-specific guideline for healthy WC. A recent meta-analysis of 29 cohorts including mainly Caucasians also reported a J-shaped association between WC and all-cause mortality, with the lowest risk at 94 cm for men and 77 cm for women [23]. This gap in the cut-off point showing the lowest mortality might be based on the differences in ethnicity, age, and BMI distribution of the study populations. Specifically, we found that the association between increased WC and higher mortality tended to be more profound among women with a normal BMI than women with an obese BMI. Likewise, de Hollander et al. [23] reported that someone with a large WC in the healthy weight category has a higher relative risk for mortality than someone with the same WC in the overweight category. These consistent findings might be explained by the increased proportion of hazardous visceral abdominal fat in the subjects with a healthy weight combined with a large WC [7]. Therefore, although the current guidelines of the American Association of Clinical Endocrinologists/American College of Endocrinology recommend only measuring WC in overweight or obese people [24], it would certainly be important to measure WC in combination with BMI in all patients and especially in subjects with a normal BMI.

Although human body composition and adiposity continuously change according to age in both men and women, there have been very few studies focusing on the differential association between WC and mortality stratified by age group. The present study showed that a larger WC than current cut-off points for abdominal obesity was positively associated with higher mortality in both groups, those aged < 60 and those aged ≥ 60 years, and a higher WC had a more detrimental impact on the risk of mortality in normal-weight and obese women aged < 60 years than in those aged ≥ 60 years. Similar to our results, in a pooled analysis of 11 cohort studies with more than 650,000 white adults, Cerhan et al. [8] found a strong positive association between WC (5-cm increments) and total mortality after accounting for BMI, and the magnitude of risk was much higher for those in the younger age group. In young individuals, obesity is primarily a troublesome risk for cardiometabolic disorders, but the function of fat mass as nutritional reserves becomes more important in advanced age; indeed, the elderly are faced with more competing risk factors for death [25]. Previously, Jee et al. [26] reported that a relative increase in the risk of death due to a high BMI was observed among subjects younger than 50 years, but not for those 65 years or older at baseline. However, our results emphasized the importance of maintaining a lower WC than the current cut-off value for abdominal obesity in both groups, those aged < 60 or those aged ≥ 60 years, although this was more critical in normal-weight and obese women aged < 60 years. In the present study, the HRs for mortality of increased WC more than 100 cm was markedly increased in normal-weight women compared to normal-weight men (HR 2.00; 95% CI 1.47–2.72 vs HR 1.33; 95% CI 1.07–to 1.64), especially for normal-weight women aged < 60 years compared with men (HR 4.76; 95% CI 1.78–12.82 vs HR 1.81; 95% CI 1.03–3.19). These results suggested that the WC might have a greater influence on the mortality of normal-weight women than on that of men, especially in young individuals. Although we could not clarify the underlying reasons, it might be partly accounted for by a positive and strong association between obesity and sex-specific cancer [27]. In Korea, the mortality risk from breast cancer is significantly higher for patients who are overweight or obese [28], and this risk increases sharply in young women [29]. Further studies should be conducted to clarify the underlying causes of this sexual dimorphism.

The present study has several limitations. First, the average follow-up duration of this study was just 5.3 years (time at risk), a relatively short period to monitor obesity-related mortality. A longer duration of follow-up decreases the influence of confounding factors of pre-diagnostic weight loss on the association between obesity and mortality. Therefore, to preclude underlying comorbidities from interfering with our results, we excluded the subjects who died within 1 year after follow-up and adjusted for the CCI. The CCI, which was developed in 1987 [30], successfully captures the effect of comorbidity burden on mortality [31]. In the present study, after adjusting for baseline CCI, although it was just scored as 0 or 1, the positive association between increased WC and mortality remained significant. Furthermore, the third-order interaction among WC categories (8 groups), BMI categories (4 groups), and baseline CCI values (0 or 1) were not statistically significant for both men and women (*p* value = 0.556 in men and 0.960 in women, respectively), meaning that the association of WC ranges with mortality according to BMI categories was not different between the subjects with and without comorbidities. The second limitation of our study is the lack of analysis on cause-specific mortality. Generally, the risk of death from atherosclerotic cardiovascular disease and cancer was higher among obese subjects, whereas the risk of death from respiratory disease was higher in underweight individuals [26]. We are currently building upon these ideas and exploring the differential relationship between WC and cause-specific mortality using this dataset. Lastly, we could not adjust all the confounding factors which could affect mortality such as economic status and family history for specific diseases. Furthermore, we could not acquire data for immigrants, even though they might be a very minor portion of our 8,796,759 study subjects. However, the strengths of our study are that we analyzed a very large-scale nationwide database using direct measures of anthropometric variables and not participants’ self-reported metrics. Furthermore, we adjusted for lifestyle habits such as regular exercise, alcohol and smoking history, well-known related factors for the change of body composition [32].

## Conclusions

The present study reinforced the critical role of central obesity in death, demonstrating a J-shaped or U-shaped relationship between WC and mortality risk after adjusting for confounding factors. The association between WC and mortality and the optimal cut-off range of WC for the lowest mortality was different according to age, sex, and BMI category. Therefore, clinicians should be aware of the usefulness of WC in addition to BMI for the assessment of obesity-related complications and mortality, and individualized cut-off points for defining a healthy WC according to age, sex, and BMI category will be necessary.

## Additional file

Additional file 1: **Table S1.** Baseline characteristics of the study subjects according to WC groups. **Table S2.** Baseline characteristics of the study subjects according to BMI groups. **Table S3.** Baseline characteristics of the study subjects according to age groups. **Table S4.** Hazard ratios (95% confidence intervals) for mortality according to WC categories stratified by BMI categories and age groups. (DOC 239 kb)
