# Supplementary material for: Differential relationship between waist circumference and mortality according to age, sex, and body mass index in Korean with age of 30–90 years; a nationwide health insurance database study
Source: BMC Med. 2018 Aug 10;16:131. doi: 10.1186/s12916-018-1114-7 (PMC6085614; doi:10.1186/s12916-018-1114-7)
Supplement: Supplementary file 1 — Table S1. Baseline characteristics of the study subjects according to WC groups. Table S2. Baseline characteristics of the study subjects according to BMI groups. Table S3. Baseline characteristics of the study subjects according to age groups. Table S4. Hazard ratios (95% confidence intervals) for mortality according to WC categories stratified by BMI categories and age groups. (DOC 239 kb) [file 12916_2018_1114_MOESM1_ESM.doc]

**Table S1. *Baseline characteristics of the study subjects according to WC* *groups***

|  | | < 70 cm | | | 70-75 cm | | | 75-80 cm | | | 80-85 cm | | | 85-90 cm | | | 90-95 cm | | | 95-100 cm | | | ≥ 100 cm | | | p-value | | |  |
| --- | --- | --- | --- | --- | --- | --- | --- | --- | --- | --- | --- | --- | --- | --- | --- | --- | --- | --- | --- | --- | --- | --- | --- | --- | --- | --- | --- | --- | --- |
| **Men** | |  | | |  | | |  | | |  | | |  | | |  | | |  | | |  | | |  | | |  |
| Age (years) | | 47.6±14.0 | | | 46.1±12.7 | | | 46.2±12.0 | | | 47.3±11.8 | | | 48.5±11.8 | | | 49.5±12.1 | | | 49.7±12.5 | | | 48.8±13.1 | | | <0.001 | | |  |
| BMI (kg/m^2^) | | 19.1±1.9 | | | 20.6±1.7 | | | 22.1±1.7 | | | 23.6±1.8 | | | 25.1±1.9 | | | 26.5±2.0 | | | 28.1±2.1 | | | 30.5±2.8 | | | <0.001 | | |  |
| Systolic BP (mm Hg) | | 118.6±14.3 | | | 120.3±13.9 | | | 122.2±13.8 | | | 124.5±13.9 | | | 126.4±14.1 | | | 128.4±14.3 | | | 130.0±14.6 | | | 132.5±15.1 | | | <0.001 | | |  |
| Diastolic BP (mm Hg) | | 74.4±9.5 | | | 75.3±9.4 | | | 76.6±9.4 | | | 78.0±9.5 | | | 79.2±9.7 | | | 80.4±9.9 | | | 81.4±10.2 | | | 83.0±10.7 | | | <0.001 | | |  |
| FBS (mg/dL) | | 94.4±23.8 | | | 95.2±23.2 | | | 97.0±23.9 | | | 99.4±25.5 | | | 101.9±27.2 | | | 104.3±29.0 | | | 106.7±31.2 | | | 110.0±34.3 | | | <0.001 | | |  |
| TC (mg/dL) | | 181.3±36.8 | | | 185.6±37.1 | | | 191.2±38.5 | | | 196.6±40.3 | | | 200.0±42.7 | | | 201.6±42.2 | | | 202.8±44.3 | | | 203.7±47.4 | | | <0.001 | | |  |
| HDL-C (mg/dL) | | 61.0±37.6 | | | 59.0±33.6 | | | 56.1±31.7 | | | 53.5±30.0 | | | 51.6±32.2 | | | 50.4±30.8 | | | 49.6±30.9 | | | 49.1±28.8 | | | <0.001 | | |  |
| LDL-C (mg/dL) | | 108.8±171.2 | | | 112.1±166.0 | | | 115.8±152.7 | | | 117.9±128.0 | | | 119.1±128.1 | | | 118.7±124.9 | | | 118.4±122.5 | | | 118.4±119.3 | | | <0.001 | | |  |
| AST(IU) | | 26.3±29.2 | | | 26.3±29.6 | | | 26.4±24.0 | | | 27.1±27.7 | | | 28.2±27.8 | | | 29.8±29.3 | | | 31.6±27.0 | | | 34.6±28.8 | | | <0.001 | | |  |
| ALT(IU) | | 21.4±32.0 | | | 22.6±27.5 | | | 24.9±25.0 | | | 28.4±33.7 | | | 32.0±29.7 | | | 35.9±31.5 | | | 40.0±33.6 | | | 46.3±41.6 | | | <0.001 | | |  |
| Alcohol (%) | |  | | |  | | |  | | |  | | |  | | |  | | |  | | |  | | |  | | |  |
| Never | | 42.9 | | | 36.3 | | | 33.3 | | | 32.0 | | | 31.8 | | | 32.2 | | | 32.8 | | | 34.0 | | | <0.001 | | |  |
| 1-2 servings/week | | 39.0 | | | 43.6 | | | 45.6 | | | 46.0 | | | 45.1 | | | 43.3 | | | 42.2 | | | 41.7 | | | <0.001 | | |  |
| ≥3 servings/week | | 18.1 | | | 20.1 | | | 21.1 | | | 22.1 | | | 23.2 | | | 24.5 | | | 25.1 | | | 24.3 | | | <0.001 | | |  |
| Smoking (%) | |  | | |  | | |  | | |  | | |  | | |  | | |  | | |  | | |  | | |  |
| Never | | 31.5 | | | 29.8 | | | 29.9 | | | 30.1 | | | 30.0 | | | 30.1 | | | 29.9 | | | 29.9 | | | <0.001 | | |  |
| Ever | | 17.6 | | | 20.5 | | | 24.1 | | | 27.2 | | | 29.2 | | | 29.9 | | | 29.8 | | | 27.7 | | | <0.001 | | |  |
| Current | | 50.9 | | | 49.8 | | | 46.0 | | | 42.8 | | | 40.9 | | | 40.0 | | | 40.4 | | | 42.4 | | | <0.001 | | |  |
| Exercise (%) | |  | | |  | | |  | | |  | | |  | | |  | | |  | | |  | | |  | | |  |
| Never | | 59.4 | | | 54.4 | | | 51.3 | | | 49.8 | | | 50.2 | | | 51.6 | | | 52.6 | | | 54.4 | | | <0.001 | | |  |
| 1-2 sessions/week | | 24.4 | | | 26.8 | | | 28.3 | | | 29.1 | | | 29.0 | | | 28.2 | | | 27.9 | | | 27.4 | | | <0.001 | | |  |
| ≥3 sessions /week | | 16.3 | | | 18.8 | | | 20.4 | | | 21.1 | | | 20.9 | | | 20.2 | | | 19.5 | | | 18.3 | | | <0.001 | | |  |
| CCI score with 0 (%) | | 28.3 | | | 28.3 | | | 26.7 | | | 23.9 | | | 21.0 | | | 18.8 | | | 17.4 | | | 16.9 | | | <0.001 | | |  |
| **Women** |  | | |  | | |  | | |  | | |  | | |  | | |  | | |  | | |  | | |  | |
| Age (years) | | | 44.6±10.4 | | | 48.4±10.7 | | | 51.9±11.1 | | | 55.1±11.3 | | | 57.2±11.4 | | | 58.6±11.5 | | | 58.9±11.8 | | | 58.1±12.5 | | | <0.001 | | |
| BMI (kg/m^2^) | | | 20.2±1.8 | | | 22.0±1.8 | | | 23.4±1.9 | | | 24.8±2.1 | | | 26.2±2.3 | | | 27.8±2.5 | | | 29.4±2.8 | | | 32.1±3.6 | | | <0.001 | | |
| Systolic BP (mm Hg) | | | 114.2±13.8 | | | 117.9±14.6 | | | 121.3±15.3 | | | 124.7±15.7 | | | 127.2±15.9 | | | 129.3±16.0 | | | 130.9±16.1 | | | 132.9±16.7 | | | <0.001 | | |
| Diastolic BP (mm Hg) | | | 71.4±9.4 | | | 73.3±9.7 | | | 75.1±9.9 | | | 76.7±10.0 | | | 78.0±10.1 | | | 79.1±10.2 | | | 80.1±10.4 | | | 81.4±10.7 | | | <0.001 | | |
| FBS (mg/dL) | | | 90.7±15.1 | | | 93.2±17.6 | | | 95.9±20.7 | | | 98.9±23.7 | | | 101.7±26.2 | | | 104.6±29.1 | | | 107±31.2 | | | 111.4±35.5 | | | <0.001 | | |
| TC (mg/dL) | | | 188.1±37.6 | | | 195.3±39.9 | | | 200.9±42.0 | | | 205.0±43.5 | | | 207.3±45.5 | | | 208.5±45.1 | | | 209.0±46.7 | | | 209.42±43.8 | | | <0.001 | | |
| HDL-C (mg/dL) | | | 63.7±32.8 | | | 60.8±34.6 | | | 58.8±35.4 | | | 57.2±36.2 | | | 56.1±35.4 | | | 55.5±33.7 | | | 55.1±32.9 | | | 54.88±32.5 | | | <0.001 | | |
| LDL-C (mg/dL) | | | 112.1±133.1 | | | 118.2±105.3 | | | 122.5±85.9 | | | 125.1±81.5 | | | 126.4±79.2 | | | 126.7±79.4 | | | 126.4±81.6 | | | 126.27±84.2 | | | <0.001 | | |
| AST (IU) | | | 21.8±24.9 | | | 22.6±19.3 | | | 23.5±20.0 | | | 24.5±24.2 | | | 25.5±23.2 | | | 26.6±22.1 | | | 27.6±20.6 | | | 29.1±20.4 | | | <0.001 | | |
| ALT (IU) | | | 17.0±22.8 | | | 18.78±21.3 | | | 20.70±22.4 | | | 22.7±22.7 | | | 24.5±22.5 | | | 26.4±24.1 | | | 28.3±37.3 | | | 30.75±26.0 | | | <0.001 | | |
| Alcohol (%) | | |  | | |  | | |  | | |  | | |  | | |  | | |  | | |  | | |  | | |
| Never | | | 73.1 | | | 75.6 | | | 78.7 | | | 81.3 | | | 82.9 | | | 83.7 | | | 83.9 | | | 83.6 | | | <0.001 | | |
| 1-2 servings/week | | | 23.5 | | | 20.8 | | | 17.9 | | | 15.5 | | | 13.9 | | | 13.3 | | | 13.0 | | | 13.2 | | | <0.001 | | |
| ≥3 servings/week | | | 3.4 | | | 3.6 | | | 3.5 | | | 3.2 | | | 3.2 | | | 3.04 | | | 3.2 | | | 3.2 | | | <0.001 | | |
| Smoking (%) | | |  | | |  | | |  | | |  | | |  | | |  | | |  | | |  | | |  | | |
| Never | | | 94.5 | | | 95.5 | | | 95.8 | | | 95.9 | | | 95.7 | | | 95.3 | | | 94.8 | | | 93.6 | | | <0.001 | | |
| Ever | | | 1.9 | | | 1.6 | | | 1.4 | | | 1.4 | | | 1.4 | | | 1.6 | | | 1.8 | | | 2.2 | | | <0.001 | | |
| Current | | | 3.6 | | | 3.0 | | | 2.8 | | | 2.7 | | | 2.9 | | | 3.0 | | | 3.4 | | | 4.2 | | | <0.001 | | |
| Exercise (%) | | |  | | |  | | |  | | |  | | |  | | |  | | |  | | |  | | |  | | |
| Never | | | 61.3 | | | 60.7 | | | 62.1 | | | 64.3 | | | 66.7 | | | 68.8 | | | 70.2 | | | 72.1 | | | <0.001 | | |
| 1-2 sessions/week | | | 21.5 | | | 20.3 | | | 18.6 | | | 17.0 | | | 15.7 | | | 14.9 | | | 14.5 | | | 14.0 | | | <0.001 | | |
| ≥3 sessions/week | | | 17.2 | | | 19.0 | | | 19.3 | | | 18.7 | | | 17.6 | | | 16.3 | | | 15.4 | | | 13.9 | | | <0.001 | | |
| CCI score with 0 (%) | | | 19.2 | | | 15.7 | | | 12.8 | | | 10.1 | | | 8.3 | | | 7.1 | | | 6.4 | | | 6.3 | | | <0.001 | | |

Data are expressed as mean ±SD for continuous variables and as percentages for categorical variables. For continuous variables, differences among the groups were tested using the one-way ANOVA. For categorical variables, differences among the groups were analyzed with the Pearson’s Chi-square test or the Fisher’s exact test. Charlson Comorbidity Index score, 0 means that the subject is without any comorbidity.

P-for linear trends for all variables (contrast test in ANOVA for continuous variables and Mantel-Haenszel χ2 test for categorical variables) were statistically significant (p-value < 0.001).

WC, waist circumference; BMI, body mass index; BP, blood pressure; FBS, fasting blood glucose; TC, total cholesterol; HDL-C, high-density lipoprotein cholesterol; LDL-C, low-density lipoprotein cholesterol; AST, aspartate aminotransferase; ALT, alanine aminotransferase; CCI, Charlson Comorbidity Index

**Table S2.** *Baseline characteristics of the study subjects according to BMI groups*

|  | BMI < 18.5 (kg/m^2^) | 18.5 ≤BMI < 23 | 23 ≤BMI < 25 | 25 ≤BMI | p-value |
| --- | --- | --- | --- | --- | --- |
| Men |  |  |  |  |  |
| Age (years) | 51.6±15.1 | 48.4±12.9 | 48.1±11.9 | 46.90±11.5 | < 0.001 |
| WC (cm) | 70.5±5.3 | 78.0±5.2 | 83.6±4.6 | 90.0±6.1 | < 0.001 |
| Systolic BP (mm Hg) | 119.6±15.3 | 122.3±14.3 | 124.9±13.9 | 128.0±14.2 | < 0.001 |
| Diastolic BP (mm Hg) | 74.8±9.9 | 76.4±9.5 | 78.1±9.5 | 80.4±9.9 | < 0.001 |
| FBS (mg/dL) | 96.9±30.0 | 97.9±26.0 | 100.3±26.1 | 103.1±27.6 | < 0.001 |
| TC (mg/dL) | 179.4±39.3 | 190.1±39.3 | 197.4±41.2 | 202.3±42.2 | < 0.001 |
| HDL-C (mg/dL) | 61.6±40.9 | 56.6±32.9 | 52.8±29.7 | 50.4±30.8 | < 0.001 |
| LDL-C (mg/dL) | 104.1±154.7 | 113.8±139.7 | 118.8±132.9 | 119.9±133.6 | < 0.001 |
| AST(IU) | 29.1±38.2 | 26.6±28.7 | 27.0±27.1 | 29.8±26.3 | < 0.001 |
| ALT(IU) | 22.1±29.9 | 24.5±31.3 | 28.7±28.5 | 36.7±32.3 | < 0.001 |
| Alcohol (%) |  |  |  |  | < 0.001 |
| Never | 42.3 | 34.7 | 32.4 | 31.2 |  |
| 1-2 servings/week | 34.8 | 42.8 | 45.4 | 46.1 |  |
| ≥3 servings/week | 22.9 | 22.5 | 22.2 | 22.7 |  |
| Smoking (%) |  |  |  |  | < 0.001 |
| Never | 27.2 | 29.5 | 30.8 | 30.1 |  |
| Ever | 17.2 | 23.6 | 28.6 | 29.1 |  |
| Current | 55.6 | 46.9 | 40.7 | 40.9 |  |
| Exercise (%) |  |  |  |  | < 0.001 |
| Never | 63.2 | 54.4 | 50.0 | 48.9 |  |
| 1-2 sessions/week | 22.5 | 26.9 | 28.6 | 29.9 |  |
| ≥ 3 sessions/week | 14.3 | 18.7 | 21.4 | 21.2 |  |
| CCI score with 0 (%) | 23.5 | 24.5 | 22.6 | 21.6 | < 0.001 |
| Women |  |  |  |  |  |
| Age (years) | 47.1±14.9 | 49.0±11.9 | 52.9±11.2 | 54.8±11.2 | < 0.001 |
| WC (cm) | 65.1±5.3 | 72.1±5.6 | 78.3±5.4 | 85.6±7.1 | < 0.001 |
| Systolic BP (mm Hg) | 113.7±14.8 | 117.4±14.9 | 122.3±15.4 | 126.9±15.8 | < 0.001 |
| Diastolic BP (mm Hg) | 71.1±9.6 | 72.9±9.8 | 75.5±10.0 | 78.1±10.1 | < 0.001 |
| FBS (mg/dL) | 91.5±19.3 | 93.4±19.1 | 96.9±21.8 | 101.1±25.3 | < 0.001 |
| TC (mg/dL) | 185.7±36.9 | 194.1±40.5 | 202.3±42.4 | 206.4±44.1 | < 0.001 |
| HDL-C (mg/dL) | 64.5±33.4 | 61.3±35.0 | 58.1±33.8 | 56.5±35.4 | < 0.001 |
| LDL-C (mg/dL) | 108.4±123.4 | 116.9±107.6 | 123.6±93.4 | 126.0±85.1 | < 0.001 |
| AST(IU) | 22.6±33.9 | 22.6±22.4 | 23.7±19.6 | 25.5±22.1 | < 0.001 |
| ALT(IU) | 16.8±30.8 | 18.4±21.4 | 21.1±21.5 | 25.0±34.9 | < 0.001 |
| Alcohol (%) |  |  |  |  | < 0.001 |
| Never | 76.9 | 76.1 | 79.3 | 81.2 |  |
| 1-2 servings/week | 19.9 | 20.4 | 17.4 | 15.7 |  |
| ≥3 servings/week | 3.2 | 3.5 | 3.3 | 3.2 |  |
| Smoking (%) |  |  |  |  | < 0.001 |
| Never | 92.4 | 95.0 | 96.1 | 95.9 |  |
| Ever | 2.2 | 1.7 | 1.4 | 1.4 |  |
| Current | 5.5 | 3.3 | 2.6 | 2.7 |  |
| Exercise (%) |  |  |  |  | < 0.001 |
| Never | 67.5 | 62.1 | 62.5 | 65.0 |  |
| 1-2 sessions/week | 19.6 | 19.9 | 18.1 | 16.7 |  |
| ≥ 3 sessions/week | 12.9 | 18.0 | 19.5 | 18.3 |  |
| CCI score with 0 (%) | 17.7 | 15.6 | 12.1 | 9.7 | < 0.001 |

Data are expressed as mean ±SD for continuous variables and as percentages for categorical variables. For continuous variables, differences among the groups were tested using the one-way ANOVA. For categorical variables, differences among the groups were analyzed with the Pearson’s Chi-square test or the Fisher’s exact test. Charlson Comorbidity Index score, 0 means that the subject is without any comorbidity. P-for linear trends for all variables (contrast test in ANOVA for continuous variables and Mantel-Haenszel χ2 test for categorical variables) were statistically significant (p-value <0.001).

WC, waist circumference; BMI, body mass index; BP, blood pressure; FBS, fasting blood glucose; TC, total cholesterol; HDL-C, high-density lipoprotein cholesterol; LDL-C, low-density lipoprotein cholesterol; AST, aspartate aminotransferase; ALT, alanine aminotransferase; CCI, Charlson Comorbidity Index

***Table S3.*** *Baseline characteristics of the study subjects according to age group*s

|  | Age < 60 years | Age ≥ 60 years | p-value |
| --- | --- | --- | --- |
| Men |  |  |  |
| Age (years) | 43.3±8.2 | 67.2±5.6 | <0.001 |
| BMI (kg/m^2^) | 24.3±3.0 | 23.7±2.9 | <0.001 |
| WC (cm) | 83.8±7.5 | 84.8±8.0 | <0.001 |
| Systolic BP (mm Hg) | 124.1±13.8 | 129.6±16.0 | <0.001 |
| Diastolic BP (mm Hg) | 78.4±9.8 | 78.6±10.1 | <0.001 |
| FBS (mg/dL) | 99.5±26.2 | 104.9±29.1 | <0.001 |
| TC (mg/dL) | 197.9±40.5 | 190.4±44.2 | <0.001 |
| HDL-C (mg/dL) | 53.1±29.7 | 53.99±38.7 | <0.001 |
| LDL-C (mg/dL) | 118.5±146.4 | 112.2±75.4 | <0.001 |
| AST(IU) | 28.0±27.6 | 28.2±27.9 | <0.001 |
| ALT(IU) | 31.3±32.1 | 25.5±27.7 | <0.001 |
| Alcohol (%) |  |  | <0.001 |
| Never | 29.0 | 49.6 |  |
| 1-2 servings/week | 49.0 | 25.8 |  |
| ≥ 3 servings/week | 22.0 | 24.6 |  |
| Smoking (%) |  |  | <0.001 |
| Never | 27.3 | 41.8 |  |
| Ever | 25.9 | 31.4 |  |
| Current | 46.8 | 26.8 |  |
| Exercise (%) |  |  | <0.001 |
| Never | 49.3 | 59.8 |  |
| 1-2 sessions/week | 31.4 | 15.7 |  |
| ≥ 3 sessions/week | 19.3 | 24.5 |  |
| CCI score with 0 (%) | 26.4 | 7.5 | <0.001 |
| Women |  |  |  |
| Age (years) | 46.1±7.6 | 67.9±5.8 | <0.001 |
| BMI (kg/m2) | 23.3±3.2 | 24.4±3.3 | <0.001 |
| WC (cm) | 75.9±8.2 | 81.7±8.3 | <0.001 |
| Systolic BP (mm Hg) | 118.6±14.8 | 129.3±16.3 | <0.001 |
| Diastolic BP (mm Hg) | 74.06±10.0 | 78.0±10.1 | <0.001 |
| FBS (mg/dL) | 94.6±19.8 | 102.1±26.7 | <0.001 |
| TC (mg/dL) | 197.2±41.2 | 206.4±44.8 | <0.001 |
| HDL-C (mg/dL) | 60.1±33.3 | 26.7±38.8 | <0.001 |
| LDL-C (mg/dL) | 119.4±105.5 | 125.6±75.2 | <0.001 |
| AST(IU) | 23.0±21.0 | 25.8±25.3 | <0.001 |
| ALT(IU) | 20.6±24.1 | 22.1±20.1 | <0.001 |
| Alcohol (%) |  |  | <0.001 |
| Never | 73.9 | 91.7 |  |
| 1-2 servings/week | 22.2 | 6.8 |  |
| ≥ 3 servings/week | 4.0 | 1.6 |  |
| Smoking (%) |  |  | <0.001 |
| Never | 95.1 | 96.5 |  |
| Ever | 1.7 | 1.2 |  |
| Current | 3.2 | 2.4 |  |
| Exercise (%) |  |  | <0.001 |
| Never | 60.8 | 70.2 |  |
| 1-2 sessions/week | 20.5 | 12.7 |  |
| ≥ 3 sessions/week | 18.6 | 17.2 |  |
| CCI score with 0 (%) | 16.0 | 4.4 | <0.001 |

Data are expressed as mean ±SD for continuous variables and as percentages for categorical variables. For continuous variables, differences among the groups were tested using the independent two-sample t-test. For categorical variables, differences among the groups were analyzed with the Pearson’s Chi-square test or the Fisher’s exact test. Charlson Comorbidity Index score, 0 means that the subject is without any comorbidity.

WC, waist circumference; BMI, body mass index; BP, blood pressure; FBS, fasting blood glucose; TC, total cholesterol; HDL-C, high-density lipoprotein cholesterol; LDL-C, low-density lipoprotein cholesterol; AST, aspartate aminotransferase; ALT, alanine aminotransferase; CCI, Charlson Comorbidity Index

**Table S4.** *Hazard ratios (95% confidence intervals) for mortality according to WC categories stratified by BMI categories and age groups*

|  | Age < 60 years | | | | Age ≥ 60 years | | | |
| --- | --- | --- | --- | --- | --- | --- | --- | --- |
|  | BMI < 18.5 (kg/m^2^) | 18.5 ≤ BMI < 23 | 23 ≤ BMI < 25 | 25 ≤ BMI | BMI < 18.5 | 18.5 ≤ BMI < 23 | 23 ≤ BMI < 25 | 25 ≤ BMI |
| **Men** |  |  |  |  |  |  |  |  |
| WC (cm) |  |  |  |  |  |  |  |  |
| < 70 | 1.06 (0.62, 1.84) | 0.82 (0.75, 0.90) | 0.80 (0.48, 1.32) | 1.00 (0.56, 1.81) | 0.94 (0.82, 1.09) | 0.76 (0.72, 0.80) | 1.58 (1.25, 1.99) | 1.38 (0.97, 1.96) |
| 70-75 | 1.05 (0.60, 1.81) | 0.85 (0.80, 0.90) | 0.84 (0.72, 0.98) | 1.14 (0.80, 1.63) | 0.98 (0.85, 1.13) | 0.81 (0.79, 0.84) | 1.16 (1.03, 1.31) | 1.48 (1.17, 1.88) |
| 75-80 | 1.31 (0.75, 2.28) | 0.87 (0.82, 0.92) | 0.90 (0.84, 0.95) | 1.02 (0.90, 1.16) | 1.07 (0.93, 1.25) | 0.85 (0.83, 0.88) | 0.91 (0.87, 0.97) | 1.21 (1.07, 1.36) |
| 80-85 | 1.16 (0.64, 2.07) | 0.91 (0.86, 0.96) | 0.93 (0.89, 0.98) | 0.97 (0.92, 1.03) | 1.11 (0.95, 1.30) | 0.90 (0.87, 0.92) | 0.93 (0.90, 0.96) | 1.00 (0.94, 1.05) |
| 85-90 | 1 | 1 | 1 | 1 | 1 | 1 | 1 | 1 |
| 90-95 | 0.71 (0.20, 2.50) | 1.30 (1.14, 1.48) | 1.15 (1.08, 1.23) | 1.10 (1.05, 1.14) | 1.00 (0.74, 1.36) | 1.16 (1.10, 1.22) | 1.17 (1.12, 1.21) | 1.14 (1.10, 1.18) |
| 95-100 | 1.86 (0.53, 6.54) | 1.30 (0.90, 1.86) | 1.37 (1.17, 1.60) | 1.21 (1.27, 1.34) | 1.15 (0.67, 1.98) | 1.39 (1.25, 1.54) | 1.42 (1.34, 1.51) | 1.32 (1.27, 1.37) |
| ≥100 |  | 1.81 (1.03, 3.19) | 1.83 (1.22, 2.75) | 1.80 (1.68, 1.93) | 1.90 (0.61, 5.95) | 1.24 (0.98, 1.56) | 1.91 (1.67, 2.17) | 1.86 (1.78, 1.95) |
| **Women** |  |  |  |  |  |  |  |  |
| WC (cm) |  |  |  |  |  |  |  |  |
| < 70 | 0.99 (0.32, 3.07) | 0.81 (0.73, 0.89) | 0.87 (0.71, 1.05) | 1.07 (0.68, 1.68) | 0.75 (0.67, 0.84) | 1.00 (0.96, 1.05) | 1.05 (0.89, 1.24) | 1.60 (1.26, 2.02) |
| 70-75 | 1.20 (0.38, 3.80) | 0.84 (0.76, 0.92) | 0.88 (0.80, 0.97) | 0.92 (0.78, 1.08) | 0.81 (0.72, 0.91) | 0.91 (0.87, 0.94) | 0.92 (0.85, 0.99) | 1.20 (1.05, 1.37) |
| 75-80 | 1.22 (0.36, 4.18) | 0.89 (0.81, 0.99) | 0.99 (0.91, 1.07) | 0.88 (0.80, 0.96) | 0.89 (0.78, 1.01) | 0.93 (0.89, 0.96) | 0.95 (0.91, 1.00) | 0.99 (0.93, 1.06) |
| 80-85 | 1 | 1 | 1 | 1 | 1 | 1 | 1 | 1 |
| 85-90 | 0.74 (0.08, 7.14) | 1.16 (0.93, 1.43) | 1.12 (0.99, 1.27) | 1.10 (1.02, 1.19) | 0.88 (0.71, 1.09) | 1.12 (1.06, 1.18) | 1.07 (1.02, 1.12) | 1.06 (1.10, 1.02) |
| 90-95 | 2.74 (0.28, 26.32) | 1.52 (0.91, 2.55) | 1.41 (1.10, 1.81) | 1.26 (1.15, 1.38) | 0.86 (0.61, 1.22) | 1.21 (1.11, 1.32) | 1.21 (1.14, 1.30) | 1.19 (1.14, 1.25) |
| 95-100 |  | 1.10 (0.35, 3.42) | 2.92 (1.81, 4.72) | 1.40 (1.24, 1.59) | 0.19 (0.06, 0.60) | 1.36 (1.15, 1.62) | 1.42 (1.26, 1.60) | 1.35 (1.28, 1.42) |
| ≥100 |  | 4.76 (1.78, 12.82) | 1.29 (0.18, 9.17) | 2.32 (2.02, 2.72) | 0.87 (0.33, 2.34) | 1.78 (1.29, 2.46) | 1.86 (1.45, 2.37) | 1.75 (1.64, 1.86) |
|  |  |  |  |  |  |  |  |  |

Data are expressed as the hazard ratio (95% confidence interval)

The hazard ratios (95% confidence intervals) were calculated by the Cox proportional hazards model with baseline age, alcohol, smoking and exercise status, BMI (as a continuous variable), BMI categories (4 groups), WC categories (8 groups), interaction between BMI categories and WC categories, FBS, total cholesterol, HDL-C, LDL-C, AST, ALT, and comorbidities, assessed by CCI
